# Batch and sampling time exert a larger influence on the fungal community than gastrointestinal location in model animals: A meaningful case study

**DOI:** 10.3389/fnut.2022.1021215

**Published:** 2022-11-07

**Authors:** Jiayan Li, Daiwen Chen, Bing Yu, Jun He, Zhiqing Huang, Ping Zheng, Xiangbing Mao, Hua Li, Jie Yu, Junqiu Luo, Hui Yan, Yuheng Luo

**Affiliations:** Key Laboratory for Animal Disease-Resistance Nutrition of Ministry of Education of China, Key Laboratory for Animal Disease-Resistance Nutrition and Feed of Ministry of Agriculture of China, Key Laboratory of Animal Disease-Resistant Nutrition of Sichuan Province, Animal Nutrition Institute, Sichuan Agricultural University, Chengdu, China

**Keywords:** model animal, gastrointestinal tract, fungi, different batch, sampling time

## Abstract

Fungi play a fundamental role in the intestinal ecosystem and health, but our knowledge of fungal composition and distribution in the whole gastrointestinal tract (GIT) is very limited. The physiological similarity between humans and pigs in terms of digestive and associated metabolic processes places, the pig in a superior position over other non-primate models. Here, we aimed to characterize the diversity and composition of fungi in the GIT of pigs. Using high-throughput sequencing, we evaluated the fungal community in different locations of GIT of 11 pigs with 128.41 ± 1.25 kg body weight acquired successively. Among them, five pigs are sacrificed in April 2019 (Batch 1) and the other six are sacrificed in January 2020 (Batch 2). All subjects with similar genetic backgrounds, housing, management, and diet. Finally, no significant difference is found in the α-diversity (Richness) of the fungal community among all intestinal segments. Basidiomycota and Ascomycota are the two predominant fungal phyla, but Batch 1 harbored a notably high abundance of Basidiomycota and Batch 2 harbored a high abundance of Ascomycota. Moreover, the two batches harbored completely different fungal compositions and core fungal genera. FUNGuild (Fungal Functional Guild) analysis revealed that most of the fungal species present in the GIT are saprotroph, plant pathogen, and animal endosymbiont. Our study is the first to report that even under the same condition, large variations in fungal composition in the host GIT still occur from batch-to-batch and sampling time. The implications of our observations serve as references to the development of better models of the human gut.

## Introduction

The gastrointestinal tract (GIT) is the home of gut microbiota, with trillions of colonizing and transient microorganisms, including bacteria, archaea, fungi, viruses, and protozoa (1). Accumulating evidence indicates that microbiota plays a critical role in many physiological processes of the host, such as digestion, metabolism, immune development, and resistance to pathogens (2–5). To date, many studies have focused on the bacterial community and function in GIT, while fungi received much less attention. The fungi referred to as the “mycobiota” comprise 1–2% of the microbial biomass in the gut ecosystem of humans and monogastric animals (6). Our understanding of fungi has changed over the last decade with the advances in sequencing technologies and the deciphering of the microbiome. More and more studies have demonstrated the importance of fungi to intestinal nutrition and immune function (1, 7–9). It is noteworthy that the size of a typical fungal cell is much larger than a typical bacterial cell, suggesting that fungi may contribute to intestinal microbial metabolism and provide a substantial mass of surface area for host-microbe interactions (10).

To explore the fungal community in the GIT, increased studies have been conducted in humans and mice. Despite the large individual variations, most of the intestinal fungi are considered to belong to the phyla Ascomycota and Basidiomycota, with *Candida, Saccharomyces, and Cladosporium* spp. as the three most abundant genera in the gut of humans and mice (10–12). A Pig is regarded as an ideal animal model to study the composition and function of the microbiome in GIT due to its anatomy and physiology being highly similar to humans (13). The pig has already been used in many studies as an animal model for humans to assess the gut microbiota, due to similarities in GIT functions and anatomical structure, metabolism, and nutritional requirements, but also due to similar major bacteria phyla occurring in the GIT of pigs (Firmicutes and Bacteroidetes) (14). However, with considerable large individual variations in fungal composition, experiments are needed to assess whether pigs can serve as a good model of human gut fungi. The critical role of gut bacteria in the metabolism and health of animals is gradually revealed, but the role of gut fungi remains to be assessed. A typical role of gut fungi may be the degradation of plant cell walls, which has been shown in many studies on ruminants (15, 16). As carbohydrates are the main source of energy for humans and animals, future studies are vital to understanding the role of commensal fungi and the utilization of dietary nutrients.

In this study, we investigated the fungal community along different locations of the GIT (from the stomach to the colon) in growing-finishing pigs with similar genetic backgrounds. These pigs are fed with a basal corn-soybean diet and selected from two successive batches under the same feeding condition. A high-throughput sequencing targeting the internal transcribed spacer 1 (ITS1) and FUNGuild is used to reveal the fungal composition and potential function in the GIT. The implications of our observation are scrutinizing the efficient use of pigs in research directed to serve human needs.

## Materials and methods

All experimental procedures and animal care were performed following the Guide for the Care and Use of Laboratory Animals prepared by the Institutional Animal Care and Use Committee of Sichuan Agricultural University, and all animal protocols were approved by the Animal Care and Use Committee of Sichuan Agricultural University under permit number DKY- B20172008.

### Animal feeding management and sample collection

A total of 11 cross-bred (Duroc × Landrace × Yorkshire) growing-finishing male pigs with similar genetic backgrounds and average body weight (128.41 ± 1.25 kg) were selected. These pigs were from the same commercial supplier and successively allocated to one pen with a concrete floor in the same mechanically ventilated room in the experimental farm of the Sichuan Agricultural University for more than three months [Batch 1 (no. 1–5), Batch 2 (no.6–11)]. A basal corn-soybean diet was used and met the nutrient requirement of the National Research Council (17) for 100–135 kg pigs, and the levels of main nutrients in the diet (Supplementary Table 1) were analyzed using the standard method recommended by the Association of Official Analytical Chemists (18). The diet was antibiotics and growth promotors free, and no fungal growth promoters or additives were supplemented. All pigs were given *ad libitum* access to fresh water and feed, the room temperature was maintained at 25–28°C and relative humidity was controlled at 55–65%.

Sampling was performed in April 2019 (Batch 1) and January 2020 (Batch 2). The pigs were humanely euthanized by being electrically stunned. The whole GIT was removed from the abdominal cavity and immediately dissected. Approximately 2 g of digesta from each of six GIT regions, the middle stomach, duodenum, jejunum, ileum, cecum, and colon, of each pig was collected into a 2-mL sterilized tube. All pigs were fasted overnight before the sampling day to ensure the homogeneity of all samples, which resulted in the failure to collect a few samples, and the sample information can be found in Supplementary Table 2. The collected samples were flash-frozen in liquid nitrogen and stored at −80°C until further processing. Samples were categorized by the GIT region: duodenum, jejunum, and ileum samples were categorized as “Upper GIT,” cecum and colon were categorized as “Lower GIT,” and the stomach remained categorized as “Stomach”.

### DNA extraction and sequencing

All samples were thawed on ice. Approximately 0.2 g of each digesta sample was added to bead-beating tubes with TE buffer, and a lysis buffer was then added for homogenization. The treated sample was then subjected to bead beating at 6.0 m/s for 40 s twice using a FastPrep-24 (MP Biomedicals, Solon, OH, USA). The genomic DNA of each sample was extracted using a QIAamp Fast DNA Stool Mini Kit (Qiagen, Hilden, Germany). DNA concentration and quality were further assessed using a NanoDrop 2000 Spectrophotometer (NanoDrop Technologies, Wilmington, DE). The ITS1 region was sequenced with specific primers (ITS1f, CTTGGTCATTTAG AGGAAGTAA; ITS2r, GCTGCGTTCTTCATCGATGC) (19–21). PCR for each sample was carried out in a 30-μL reaction with 15 μL of Phusion^®^ High-Fidelity PCR Master Mix (New England Biolabs, Massachusetts, United States), 3 μL of forward and reverse primers, and 10 μL of template DNA, as well as 2 μL of H_2_O. The thermal cycling was initiated with a denaturation step at 98°C for 1 min, followed by a sequence of 30 cycles of denaturation at 98°C for 10 s, annealing at 50°C for 30 s, elongation at 72°C for 30 s, and finalized with an elongation at 72°C for 5 min. Three parallel PCR reactions were conducted for each sample, and the PCR products were detected with electrophoresis using 2% agarose gel. PCR products for each sample were mixed in equal density ratios and the mixture was then purified with a GeneJET™ Gel Extraction Kit (Thermo Scientific, United States). Sequencing libraries were generated using an Ion Plus Fragment Library Kit 48 rxns (Thermo Scientific) following the manufacturer’s recommendations. The library quality was assessed on the Qubit^®^ 2.0 Fluorometer (Thermo Scientific). Finally, the library was sequenced on an Ion S5™ XL platform and 400 bp single-end reads were generated. To minimize the other effects (i.e., DNA extraction kits or equipment), the genomic DNA from all samples was extracted with the same batch of kits and then amplified together on an Ion S5™ XL platform.

### Bioinformatics analysis

QIIME2 (2022.2^1^) (22) was used to process and classify raw sequences. Raw reads were denoised with the DADA2 plugin in QIIME2 (23). This plugin produces fine-scale resolution through amplicon sequence variants (ASVs), and its workflow consists of filtering (e.g., primer trimming only for metabarcoding), dereplication, and reference-free chimera detection. In brief, single-end reads were assigned to samples based on their unique barcode and truncated by cutting off the barcode and primer sequences. The truncation was performed based on a quality score of 20 and with a maximum number of expected errors (maxEE) set to 2. We did not truncate the sequences to a fixed length due to the variable length of fungal ITS fragments. The sequences were clustered into ASVs and then filtered for chimeras. The UNITE database (Version 8.3^2^) was trained using the naive Bayesian classifier in QIIME2, and then the taxonomy was assigned to filtered ASVs using a pretrained UNITE database with 99% identity for fungi. The identified taxonomies were then assigned to representative sequences. Finally, the 58 digesta samples yielded a total of 2,277,899 non-chimeric sequence reads. Separate rarefaction curves for digesta samples were produced and visualized using the vegan package in R software (v 4.1.2) to determine minimum sequencing depth, and we rarefied our data to 10,000 reads per sample for downstream analysis (Supplementary Table 3).

The α-diversity of each sample was reflected by 4 indices (Richness, Chao1, Shannon, and Good’s coverage), which were calculated in QIIME2 and visualized by R (V 4.1.2). R was used to analyze the β-diversity of each sample based on multivariate statistical techniques, including principal coordinate analysis (PCoA) and non-metric multidimensional scaling (NMDS) of Bray-Curtis and unweighted UniFrac distance matrixes. Wilcoxon rank test, adonis permutational multivariate analysis of variance (also called PERMANOVA), and ANOSIM (analysis of similarities) tests were performed with R package vegan. The linear discriminant analysis effect size (LEfSe) analysis was performed using the Galaxy web application.^3^ To test the core fungal community in all samples, the compute_core_microbiome.py command in QIIME was used, and the core ASVs were required to be present in at least 80% of the samples based on the data set.

### The FUNGuild analysis

The ASVs’ taxonomic information was uploaded to the FUNGuild database for functional prediction (trophic modes and guilds) (24), and the sequences were aligned using the PhyDE (Phylogenetic Data Editor). Since the calling of guilds in the FUNGuild is predicated on the confidence of assigned taxonomy, a 93% threshold was chosen to represent a reasonable general cutoff point for ITS-based inputs in this study. For this procedure, we considered all assignments with a confidence score of “probable” or “highly probable.” Genera not represented in the database or with a confidence score of “possible” were classified as undetermined.

### Statistical analysis

Data were analyzed using R (V 4.1.2) and SPSS 23.0 statistical software (SPSS Inc., Chicago, IL, USA). α-diversity and β-diversity data were performed and visualized entirely in R using the ggplot2 package with custom R scripts. The relative abundance of fungal taxa was assessed using the Kruskal-Wallis test, and Dunn’s test was applied to conduct multiple comparisons. The linear mixed effect model (R package nlme) was used to analyze the differences in the α-diversity of the fungal community among sampling time, the GIT region, and body weight. Interactions were removed from the model if they were not significant (*p* > 0.05). Differences were considered significant when *p* < 0.05, while the differences were defined as insignificant when *p* ≥ 0.05. A significant trend was defined when 0.05 < *p* < 0.1.

## Results

### The diversity of the fungal community in the gastrointestinal tract

**FIGURE 1:**
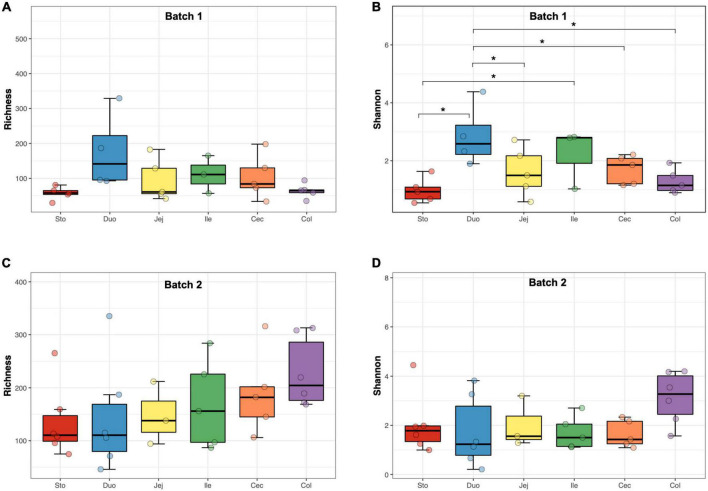
The α-diversity of the fungal community in different regions of the gastrointestinal tract in the pigs. **(A)** Richness (batch 1). **(B)** Shannon index (batch 1). **(C)** Richness (batch 2). **(D)** Shannon index (batch 2). Significance is indicated by “*” when *p* < 0.05.

There were 39,274 high-quality sequences in each sample on average, and the rarefaction curves showed that a minimum sampling depth of 10,000 sequences was sufficient to capture fungal diversity (Supplementary Figure 1). Indices for Richness, Shannon, Chao 1, and Good’s coverage were calculated to measure the α-diversity. The Good’s coverage estimates averaged 0.99, indicating a sufficient and representative sequencing depth (Supplementary Table 4). In Batch 1, the Shannon index of the fungal community in the samples from the duodenum was higher than that from other locations (*p* < 0.05, Figure 1B), while no difference in the Richness was found among the samples from different regions (Figure 1A). Meanwhile, the α-diversity of the fungal community showed no difference among diverse gastrointestinal segments in Batch 2 (Figures 1C,D). Based on the mixed model analysis, the Richness and Chao1 indices were significantly affected by sampling time (*p* < 0.01, Table 1), while the GIT region and body weight showed no significant effect on all α-diversity indices. Furthermore, an interaction between the GIT region and sampling time was detected for the Richness and Chao1 indices (*p* < 0.05).

**TABLE 1:** Linear mixed effect model analysis of fungal α-diversity.

Item	Sampling time		Region						*P-value*			
	Batch 1 (2019.4)	Batch 2 (2020.1)	Stomach	Duodenum	Jejunum	Ileum	Cecum	Colon	*p*_Time_	*p*_Region_	*p*_Weight_	*p*_Time*region_
Richness	98 ± 13^a^	171 ± 15^b^	100 ± 20	157 ± 32	114 ± 2	148 ± 27	147 ± 26	154 ± 30	0.004	0.14	0.59	0.05
shannon	1.70 ± 0.17	2.07 ± 0.20	1.56 ± 0.32	2.19 ± 0.44	1.77 ± 0.31	1.89 ± 0.28	1.68 ± 0.16	2.29 ± 0.38	0.20	0.35	0.69	0.25
Chao1	100.37 ± 12.90^a^	198.68 ± 16.54^b^	110.89 ± 22.32	174.36 ± 33.18	120.26 ± 24.17	164.50 ± 28.54	168.54 ± 31.73	176.56 ± 336.38	0.001	0.08	0.75	0.04

Data are presented as means ± standard error (SEM). Different alphabetical (a, b) superscripts mean significant difference (*p* < 0.05).

**FIGURE 2:**
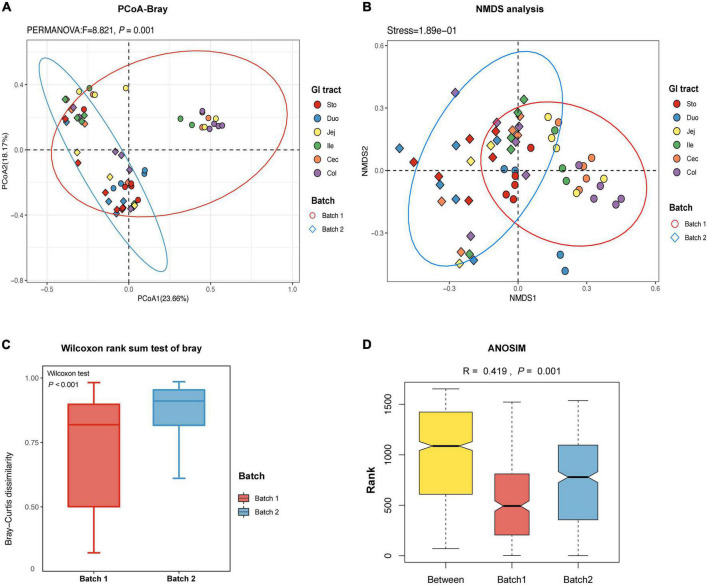
The β-diversity of fungal community in different regions of the gastrointestinal tract in the pigs. **(A)** Principal coordinate analysis (PCoA) of Bray-Curtis distances showed the stratification of batch 1 from batch 2 samples by their fungal compositional profiles. **(B)** Non-metric multidimensional scaling (NMDS) analysis showed that fungal profiles formed two clusters. A stress value less than 0.2 indicates that the model grouping is reliable. **(C)** Based on the ASVs feature table and Bray-Curtis distances, the Wilcoxon rank sum test is used to identify the significant difference and the degree of difference in fungal microbiome structure within the groups is compared. **(D)** Based on the distance index ranking, ANOSIM (analysis of similarities) confirmed that the distance between groups is significantly greater than the distance within groups, indicating that the fungal structure of the two batches is significantly different (*R* > 0, *p* < 0.05). Each point represents one sample and colors indicate different GIT regions. Sto: Stomach, Duo: Duodenum, Jej: Jejunum, Ile: Ileum, Cec: Cecum, Col: Colon.

The PCoA plot based on the Bray-Curtis (PERMANOVA: *F* = 8.821, *p* = 0.001, Figure 2A) and unweighted UniFrac (PERMANOVA: *F* = 6.456, *p* = 0.001, Supplementary Figure 2) distance matrices showed that samples from the two batches were clearly divided into two different clusters. To overcome the shortcomings of linear models (PCoA) and better reflect the non-linear structure, we evaluated the accuracy of the model with NMDS stress values. We confirmed that the stress values of the Bray index were less than 0.2 (Figure 2B), which ensured the reliability of the model. The significance was shown by the Wilcoxon rank sum test further emphasized the difference in the fungal community between the two batches (*p* < 0.001, Figure 2C). The ANOSIM test confirmed that the distance between batches was greater than the distance within batches (*R* = 0.419, *p* < 0.05, Figure 2D). In detail, samples from the stomach and duodenum were clustered to distinguish them from those samples from other intestinal segments in Batch 1 (PERMANOVA: *F* = 3.917, *p* = 0.001, Supplementary Figure 3). However, samples from different intestinal segments showed a high degree of overlap in Batch 2 (PERMANOVA: *F* = 1.331, *p* = 0.113, Supplementary Figure 3).

### The community and composition of fungi in the gastrointestinal tract

The predominant taxonomic composition of fungi across the GIT was investigated at the phyla and genera levels. According to the ASVs annotations, a total of 11 known fungal phyla (Ascomycota, Basidiomycota, Mucoromycota, Mortierellomycota, Rozellomycota, Olpidiomycota, Glomeromycota, Aphelidiomycota, Chytridiomycota, Zoopagomycota, and Kickxellomycota) were identified in all samples (Supplementary Figure 4 and Supplementary Tables 5, 6). Ascomycota and Basidiomycota were identified as the two predominant fungal phyla in the GIT, and the most abundant phylum identified in Batch 1 was Basidiomycota, but Ascomycota was the most predominant phylum in Batch 2. The abundance of phyla Ascomycota, Basidiomycota, and Mortierellomycota was different among gastrointestinal locations in Batch 1 (*p* < 0.05), and the abundance of Mucoromycota and Rozellomycota was different between the upper GIT and lower GIT in Batch 2 (*p* < 0.05). Interestingly, we found that the abundance of Basidiomycota and Mucoromycota increased gradually from the stomach and upper GIT to the lower GIT in batches 1 and 2, respectively (*p* < 0.05, Supplementary Figures 4A,B).

**FIGURE 3:**
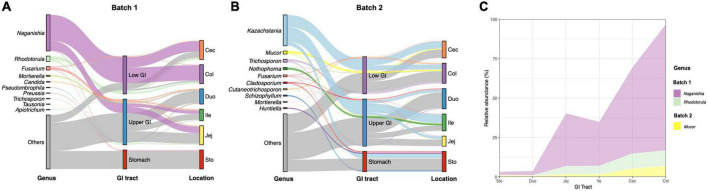
Variations of fungal composition in different regions of the gastrointestinal tract at the genus level. Relative abundance of the top 10 abundant genera over intestinal segments (stomach, duodenum, jejunum, ileum, cecum, and colon) are aggregated and colored on a Sankey-stream graph. **(A)** Batch 1, **(B)** Batch 2. Low abundance taxa (except for the 10 most abundant genera) are grouped together as “others”. Lines represent associations between indicator genera and intestinal segments, colored by genus taxa. The line width is scaled to reflect the relative abundance. **(C)** Trends in mean relative abundances of significant fungi along the GIT of growing-finishing pigs from two batches.

At the genus level, all sequences obtained from batches 1 and 2 were affiliated with 230 and 358 known fungal genera, respectively. The fungal composition of samples from all GIT locations in these pigs differs between batches. For example, in Batch 1, *Naganishia, Rhodotorula, Fusarium, Mortierella*, and *Candida* were identified as the top 5 genera across all gastrointestinal segments (Figure 3A, Supplementary Figure 4C, and Supplementary Table 5). Of these genera, the relative abundance of *Naganishia, Rhodotorula, Mortierella*, and *Candida* was different among locations (*p* < 0.05), with an increasing tendency of *Naganishia* and *Rhodotorula* from the stomach and upper GIT to the lower GIT (Figure 3C). Yet, the dominant fungal genera in the gut of pigs from Batch 2 were *Kazachstania, Mucor, Trichosporon, Nothophoma*, and *Fusarium* (Figure 3B, Supplementary Figure 4D, and Supplementary Table 6), with a difference in the relative abundance of *Mucor* among intestinal segments (Figure 3C, *p* < 0.05), which showed an increasing tendency from the stomach and upper GIT to the lower GIT.

**FIGURE 4:**
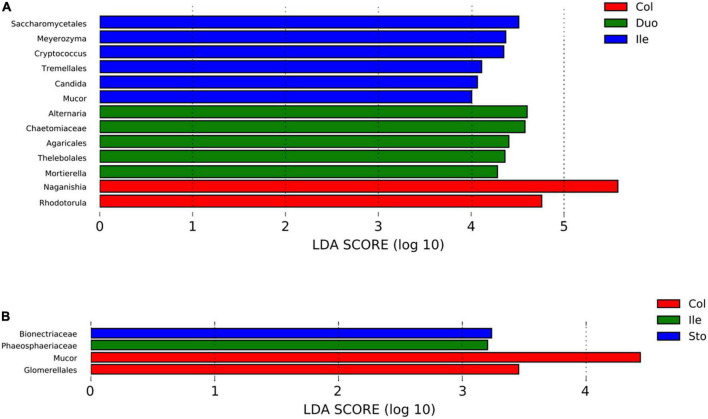
Linear discriminant analysis (LDA) effect size (LEfSe) identified the differences in fungal taxa in different regions of the gastrointestinal tract in the pigs (LDA score > 2.0, *p* < 0.05). **(A)** Batch 1, **(B)** Batch 2. Horizontal bars represent the effect size for each taxon. Sto: Stomach, Duo: Duodenum, Jej: Jejunum, Ile: Ileum, Cec: Cecum, Col: Colon.

Using LefSe analysis, we compared the specific fungal taxa in the six different gastrointestinal locations. The results revealed 13 and 4 specific fungal taxa (at genus, family, and order levels) with a significant difference among segments in pigs from Batches 1 and 2, respectively (LDA score > 2.0, *p* < 0.05). Detailly, 5 (*Alternaria, Chaetomiaceae, Agaricales, Thelebolales*, and *Mortierella*), 6 (*Saccharomycetales, Meyerozyma, Cryptococcus, Tremellales, Candida*, and *Mucor*), and 2 (*Naganishia* and *Rhodotorula*) taxa were enriched in the duodenum, ileum, and colon of pigs from Batch 1 (Figure 4A). While for those pigs from Batch 2, *Bionectriaceae* and *Phaeosphaeriaceae* showed enriched in the stomach and ileum, respectively, and *Mucor* and *Glomerellales* were enriched in the colon (Figure 4B).

### The core fungal community in the gastrointestinal tract

**FIGURE 5:**
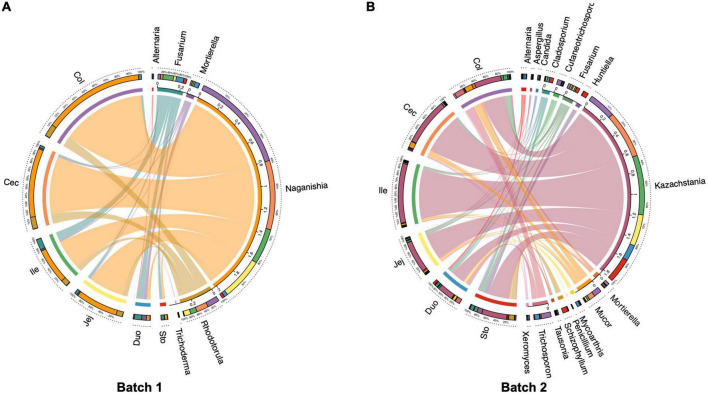
Circos plot showing the distributions of core fungal genera in different regions of the gastrointestinal tract in the pigs (present in more than 80% of all digesta samples). **(A)** Batch 1, **(B)** Batch 2.

The core fungal community for each intestinal segment was defined as those taxa found in more than 80% of samples. The core gut fungal taxa in Batch 1 consisted of 6 genera including *Naganishia, Rhodotorula, Fusarium, Mortierella, Trichosporon*, and *Alternaria* (Figure 5A). For those pigs from Batch 2, a total of 16 core fungal taxa were identified, with *Kazachstania, Mucor, Fusarium, Trichosporon*, and *Cladosporium* as the five dominant core genera (Figure 5B).

### The predicted functional composition of the fungal community

**FIGURE 6:**
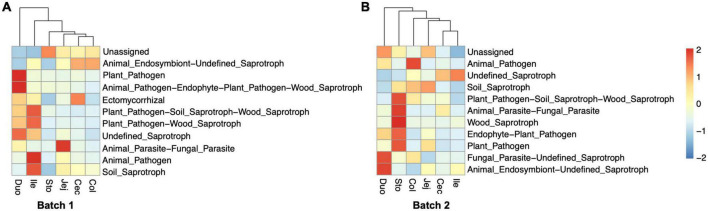
Functional prediction of fungal community in different regions of the gastrointestinal tract in the pigs. Heatmap representing the relative abundance of predicted fungal functional guilds. Trophic guild assignments for fungal sequences were analyzed based on the FUNGuild database. **(A)** Bacth1, **(B)** Batch2.

Based on the ASV’s taxonomic data, we used FUNGuild to perform the functional classification prediction of the observed fungal communities. For the samples from Batch 1, the fungal taxa were classified into eight trophic modes and 46 ecological guilds except for unassigned. The results indicated that Saprotroph and Pathotroph-Saprotroph were the representatives and dominant predicted functional trophic modes of the fungal community across all segments, with three main fungal guilds named animal endosymbiont, plant pathogens, and undefined saprotroph (Figure 6A). For those samples from Batch 2, the fungal taxa were classified into eight types (most as saprotrophs) and 49 ecological guilds, with animal pathogens, plant pathogens, and undefined saprotrophs as predominant guilds (Figure 6B). The relative abundances of functional trophic modes guilds in different gastrointestinal locations of each batch were summarized in Supplementary Tables 7, 8.

## Discussion

Accumulating evidence has shown that gut fungi have an indisputable role in host homeostasis and gut ecosystem, despite its constituting only a small proportion of gut (25, 26). Although gut microbiota research has been given too much attention, studies on fungi are constrained due to technical defects and incomplete databases. To date, little is known about fungal diversity and composition across the whole animal GIT. In addition to limited studies on gut fungi in humans and mice, the fungal community in the gut of a pig, an important model animal, has received increasing attention (27–31). In our previous studies (27, 28, 32), we focused on the fungal community in the gut of pigs with different genetic backgrounds and diets. Here, we reveal unexpected differences in the gut fungal diversity and composition along different locations of GIT between two batches of pigs as model animals.

Unlike what we expected, the difference in the fungal community among the GIT locations appears to be smaller than that between batches. Similar results are observed in chickens, batch-to-batch variations of microbiota are found in the cecum of chickens across three similar trials, represented by individually analyzed samples from 207 birds (33). Another research in two separate shipment batches of 24 Wistar rats also reports differences in the gut microbiota revealed by 16S rRNA amplicon sequencing. It is worth mentioning that these rats are obtained from the same commercial supplier and subjected to the same experimental treatment (34). A study following fungal communities in mice shows that different cages of mice receiving the same treatment also differ in their dominant fungal lineage, and their gut mycobiome varies substantially over time. These findings occur in mice housed in the same animal facility and on a homogeneous diet (35). There is research elaborated with very small numbers of human subjects, demonstrating diurnal changes in the composition of gut microbiota, and more details are described in mice (36, 37). If confirmed, such volatility of gut microbiota would necessitate protocols with standardized stool sampling times (seasons, hours of the day) to support comparisons across studies and allow generalizations to be deduced. Although we collected digesta samples from only 11 pigs, the overall comparison showed extensive differences in the fungal community between the two batches, which are consistent with the above studies. Indeed, previous studies identified that season and pen greatly influenced the gut bacterial composition of pigs (38). We have reasons to believe that different sampling times may lead to changes in the fungal community, even if other conditions are the same. The batch-to-batch variation suggests a need for large-number of samples to faithfully reflect the microbial profile investigated, even within one trial, which should be taken into account during experimental design.

Compared with gut bacteria, the biodiversity of the gut fungal community is lower and characterized by greater unevenness, which is consistent with previous studies (39–41). The diversity of gut bacteria is regarded to linearly increase along the GIT of pigs (42). However, we found that the fungal α-diversity of fungi maybe with a completely different presentation. The upper GIT (duodenum) of pigs in Batch 1 harbored higher fungal diversity than that in the hindgut, but a similar phenomenon is not been observed in Batch 2. Generally, fewer numbers and diversity of microorganisms can be found in the stomach and upper GIT compared with the hindgut due to rapid luminal flow and lower pH (43, 44). But several acid-resistant fungi have been found in the stomach, such as *Candida* and *Phialemonium* (40, 45). *Candida albicans*, one of the conditionally pathogenic fungi, is found in 43% of jejunal aspirate/culture at a threshold of at least 10^2^ CFU/mL (46), suggesting some gut fungi may be acid-tolerant. These factors may lead to the failure of investigation of clearly fewer fungi in the stomach and foregut. Fungal signaling pathways responding to external pH signals are important components of their cellular machinery. For example, *Candida albicans* is a commensal fungus that commonly colonizes mucosal areas such as the oral, GIT, and vaginal tract of human, the pH in these different organs range from acidic (the stomach and the vaginal tract) to slightly alkaline (oral-pharyngeal tract). To survive pH changes of this magnitude, *C. albicans* depends on pH-sensing pathways (Rim101 pathway) to regulate its pH tolerance (47–49). Thus, we may speculate that the high diversity of fungi in the stomach of pigs in Batch 1 may be due to its greater tolerance in highly acidic environments. However, we cannot find sufficient evidence to infer why pigs in Batch 2 were not observed significantly more fungi in the foregut than in the hindgut.

We found that the predominant fungal phyla in the GIT were Ascomycota and Basidiomycota, which is in line with the results obtained in previous studies on humans and mice (27, 28), indicating that the fungal composition in the GIT of pigs, mice, and human may be homogeneous at the phylum level. Among all classifiable fungal genera, composition and relative abundance varied significantly between the two batches. Unlike humans and mice, the commonly found fungi belonging to genera *Candida* and *Saccharomyces* are either absent or found at a low relative abundance in our study (1, 50). Instead, we found *Naganishia* is a core fungal taxon in pigs from Batch 1 with an increasing trend along the GIT. *Naganishia* (formerly *Cryptococcus*) is a commensal fungus observed in the human gut, skin, oral cavity, and scalp (51, 52). A higher relative abundance of *Naganishia* in the feces is observed in healthy Tibetan piglets when compared with the diarrhea group (53). This is a novel fungal genus known for its ability to utilize a variety of carbon sources including lignocellulosic hydrolysates, complex sugars, and fatty acids (54, 55), and its high abundance in the hindgut probably because the hindgut is the main location for fermentation of complex carbohydrates. Interestingly, the relative abundance of *Naganishia* is found extremely low in pigs from Batch 2 fed the same diet. Instead, we found a group of yeast, *Kazachstania* spp., is enriched in the gut of these pigs. Species *K. slooffiae* is found in most pigs (28, 30, 42) and may positively contribute to the body weight and gut health of piglets (56, 57). Mycobiome instability suggests transient colonization in the GIT by some fungi due to environmental and dietary exposures. While this may be the case for certain fungal species, various yeasts do stably colonize in the GIT of murine (58, 59), and longitudinal sampling could be used to identify a stable gut mycobiota that might be likely residents. More comprehensive and accurate profiling will be needed to reveal whether the fungal population in the GIT is residents or passers-by.

Different from the huge differences in fungal composition along with the GIT between the two batches, we found that the predicted function of the fungal community in the two batches of pigs may be similar. The high percentage of “undefined saprotrophs” fungal taxa may assist in the breakdown of indigestible dietary fiber and the redistribution of nutrients (60, 61). In soil, saprophytic fungi are well known for producing secondary metabolites, which play a crucial role in the initial destruction of complex organic compounds (62). Although all pigs included are healthy, there is still a small part of intestinal fungi categorized as pathogenic guilds, these fungi may serve as opportunistic pathogens. Plant pathogenic and ectomycorrhizal fungi are naturally found in plants and soil (63, 64), and their presence in the GIT of pigs may be originated from diet or soil. Compared with the upper GIT, the abundance of plant pathogenic fungi in the hindgut is almost absent, which supports the view mentioned above that food-borne fungi may not colonize or inhabit the intestinal tract in the long-term. Unlike humans and model animals, pigs have more opportunities to contact soil, and the habit of digging the earth with their snouts may be another way for the fungi in the soil to enter the pig gut (65). The abundance of animal endosymbiont increased along the GIT, indicating that the hindgut harbored more commensal fungi and can colonize or inhabit the gut for the long-term.

In summary, we provided a comprehensive overview of the fungal composition along the GIT in pig model from two separate batches, as well as new insight into potential functional profiles of gut fungi. Our results demonstrated that the most abundant phyla in the GIT were Ascomycota and Basidiomycota, and gut fungal distribution between the two batches was distinct, indicating gut fungi in healthy pigs is highly variable. In addition, even with the same diet and feeding environment, animals with similar genetic backgrounds and body weights may harbor completely different diversity and composition of fungi along the GIT. These findings emphasize that sampling time or season may be an important factor to be considered in research on intestinal fungi.

## Data availability statement

The data presented in this study are deposited in the NCBI repository (www.ncbi.nlm.nih.gov), accession number PRJNA855916.

## Ethics statement

The animal study was reviewed and approved by DC and YL, Sichuan Agricultural University. Written informed consent was obtained from the owners for the participation of their animals in this study.

## Author contributions

JiL, DC, and YL conceived and designed the experiments. DC and YL administered the project. JiL performed the experiments and collected and analyzed the data. JiL and YL wrote and revised the manuscript. BY, JH, ZH, PZ, XM, HL, JY, JuL, and HY contributed valuable advice on the data analysis and manuscript. All authors reviewed the manuscript and agreed to the published version of the manuscript.

## Footnotes

1
http://qiime.org/

2
https://doi.org/10.15156/BIO/1264708

3
https://huttenhower.sph.harvard.edu/galaxy/
