# Corrigendum: Batch and sampling time exert a larger influence on the fungal community than gastrointestinal location in model animals: A meaningful case study

**DOI:** 10.3389/fnut.2022.1126984

**Published:** 2023-01-05

**Authors:** Jiayan Li, Daiwen Chen, Bing Yu, Jun He, Zhiqing Huang, Ping Zheng, Xiangbing Mao, Hua Li, Jie Yu, Junqiu Luo, Hui Yan, Yuheng Luo

**Affiliations:** Key Laboratory for Animal Disease-Resistance Nutrition of Ministry of Education of China, Key Laboratory for Animal Disease-Resistance Nutrition and Feed of Ministry of Agriculture of China, Key Laboratory of Animal Disease-Resistant Nutrition of Sichuan Province, Animal Nutrition Institute, Sichuan Agricultural University, Chengdu, China

**Keywords:** model animal, gastrointestinal tract, fungi, different batch, sampling time

In the published article, there was an error in the author list, and author [Daiwen Chen] was erroneously excluded as corresponding author. The corrected author list appears below.

Jiayan Li, Daiwen Chen^*^, Bing Yu, Jun He, Zhiqing Huang, Ping Zheng, Xiangbing Mao, Hua Li, Jie Yu, Junqiu Luo, Hui Yan and Yuheng Luo^*^

^*^Correspondence:

Daiwen Chen

dwchen@sicau.edu.cn

Yuheng Luo

luoluo212@126.com

The authors apologize for this error and state that this does not change the scientific conclusions of the article in any way. The original article has been updated.

