# Supplementary figures and images for: Batch and sampling time exert a larger influence on the fungal community than gastrointestinal location in model animals: A meaningful case study
Source: Front Nutr. 2022 Nov 7;9:1021215. doi: 10.3389/fnut.2022.1021215 (PMC9676510; doi:10.3389/fnut.2022.1021215)

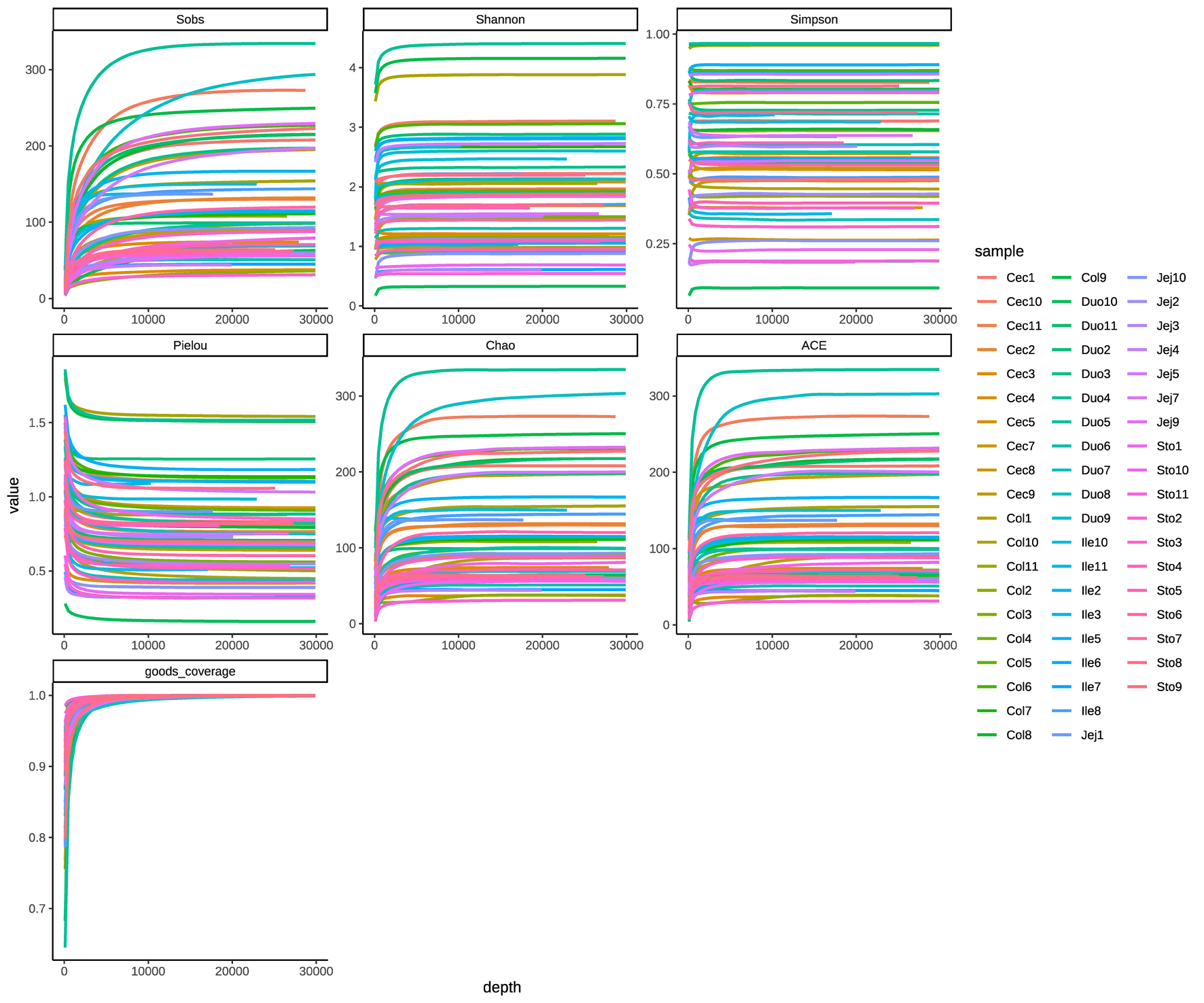

Supplement: Supplementary file 1 [file Image_1.TIF]

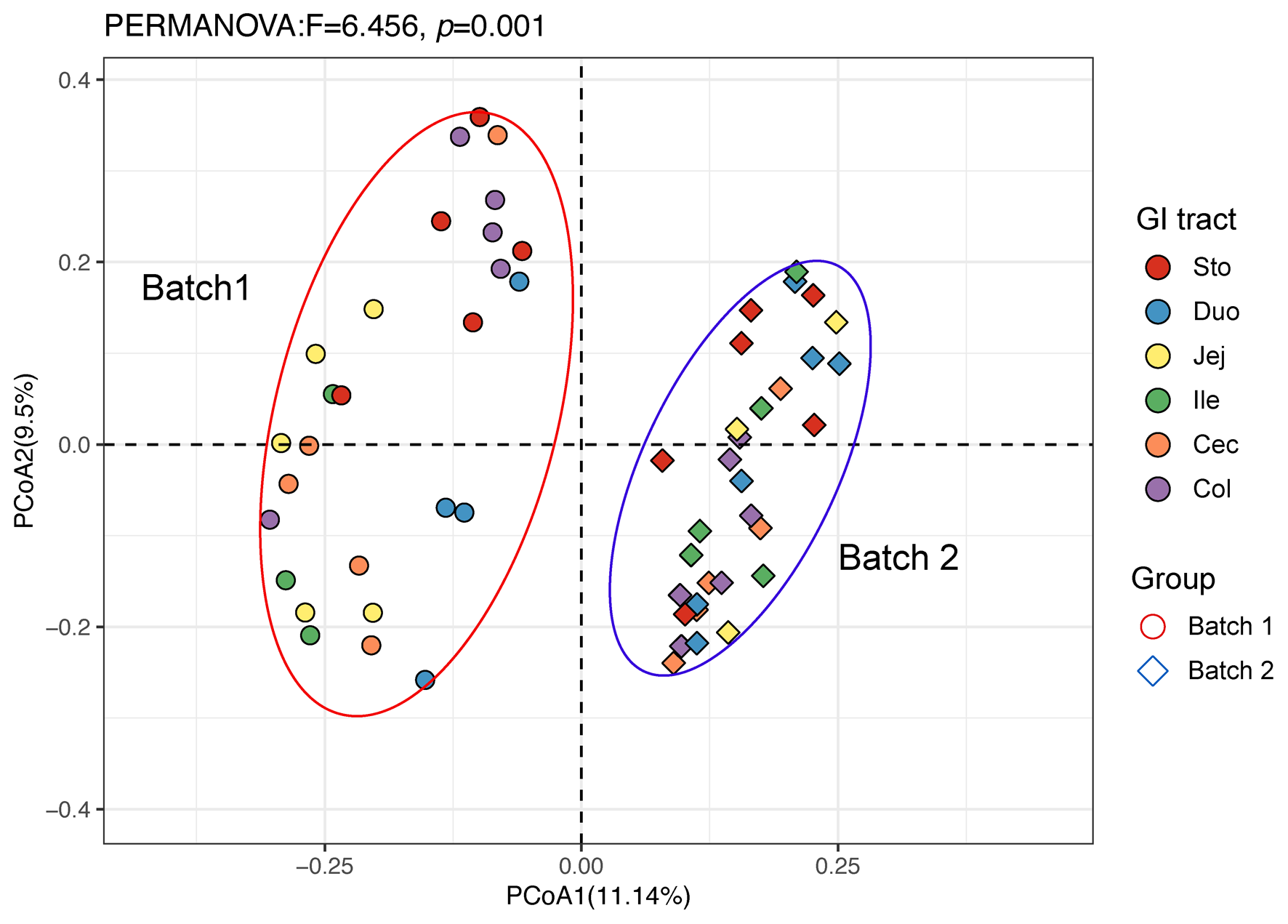

Supplement: Supplementary file 2 [file Image_2.TIF]

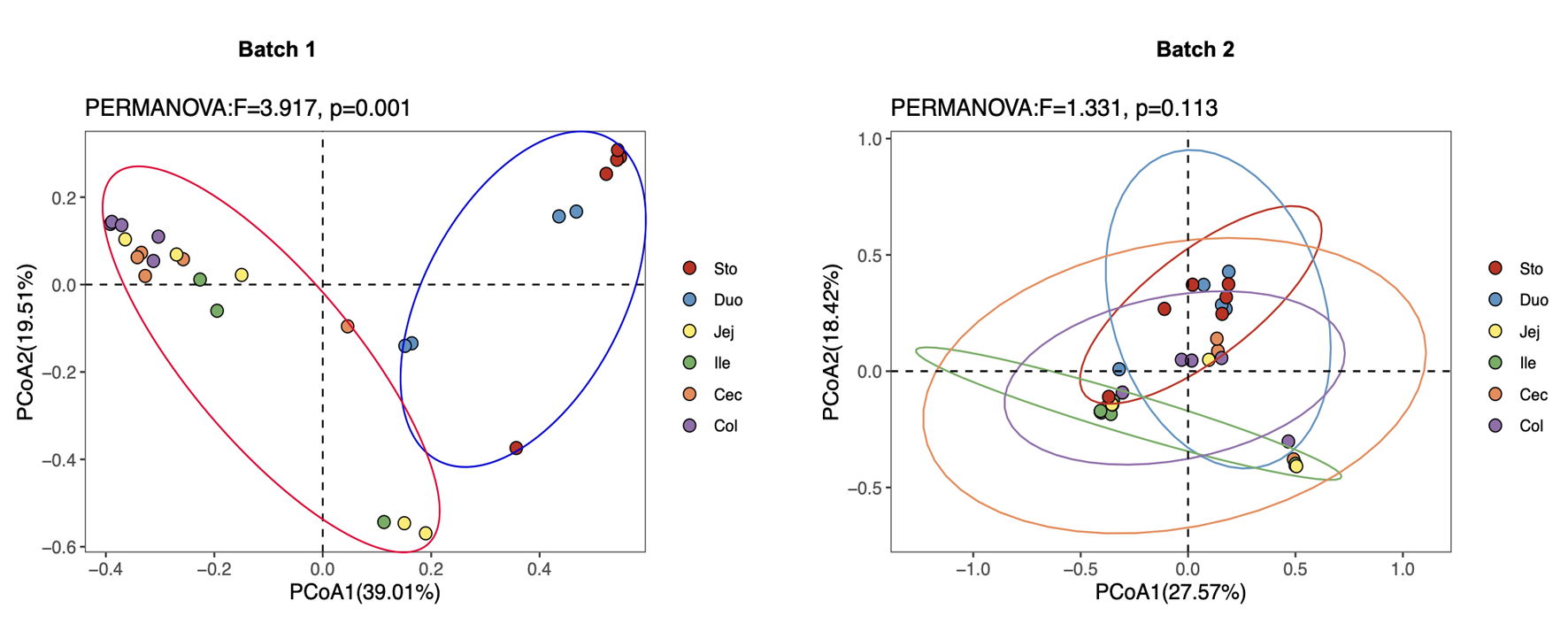

Supplement: Supplementary file 3 [file Image_3.TIFF]

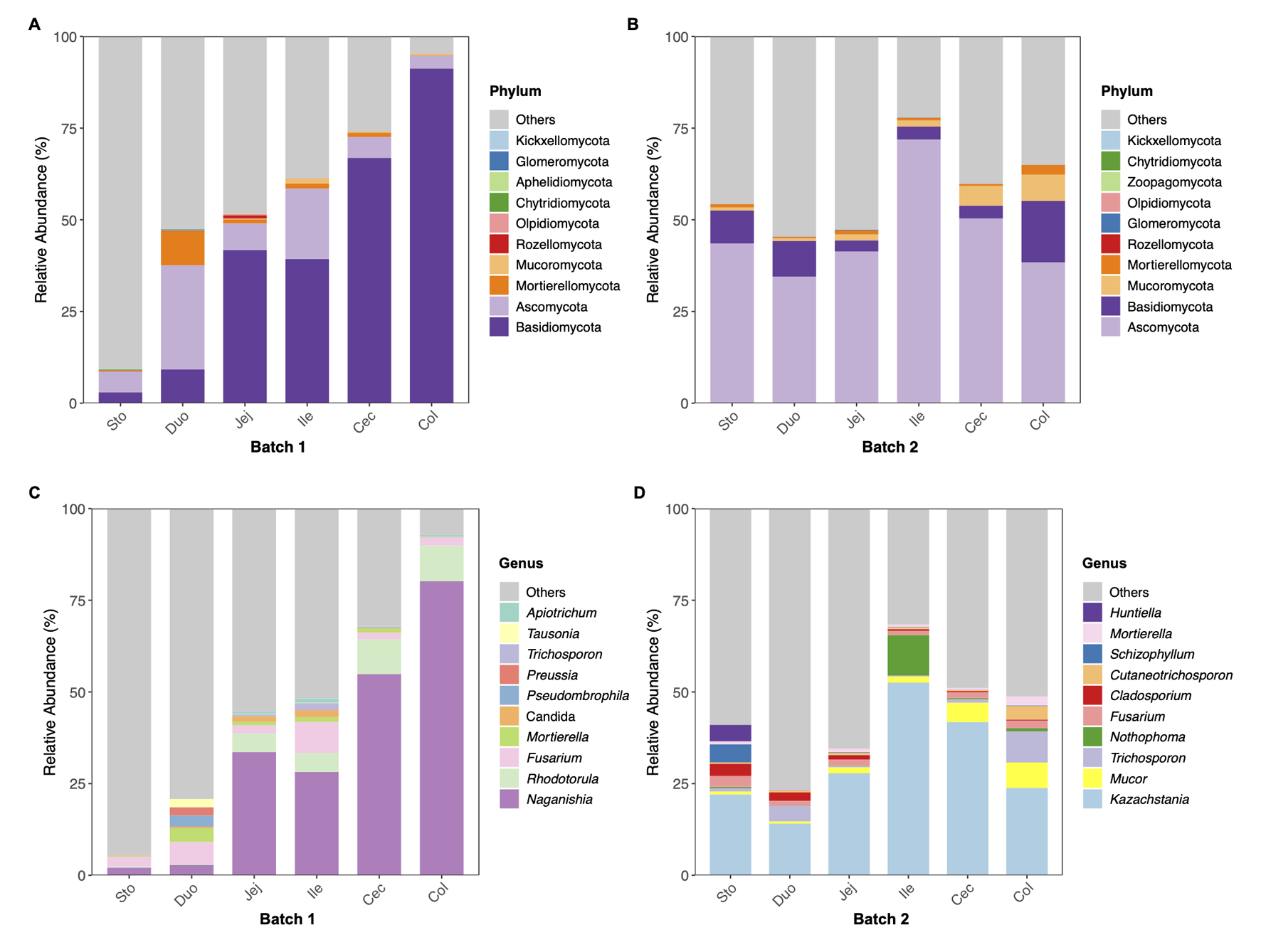

Supplement: Supplementary file 4 [file Image_4.TIFF]
