# Supplementary material for: Batch and sampling time exert a larger influence on the fungal community than gastrointestinal location in model animals: A meaningful case study
Source: Front Nutr. 2022 Nov 7;9:1021215. doi: 10.3389/fnut.2022.1021215 (PMC9676510; doi:10.3389/fnut.2022.1021215)
Supplement: Supplementary file 7 [file Data_Sheet_3.doc]

Supplementary Material

**Batch and sampling time exert a larger influence on the fungal community than gastrointestinal location in model animals: a meaningful case study**

**Supplementary Table 1** The level of main nutrients in the diets of two batches

| Item | Batch 1 | Batch 2 |
| --- | --- | --- |
| Crude protein(%) | 11.48 | 10.52 |
| Dietary energy (Kcal/Kg) | 3.38 | 3.41 |
| Ca（%） | 0.48 | 0.47 |
| Total P(%) | 0.43 | 0.41 |
| SID Lys, % | 0.64 | 0.66 |
| SID Met, % | 0.22 | 0.19 |
| SID Thr, % | 0.42 | 0.41 |
| SID Trp, % | 0.12 | 0.12 |

The level of main nutrients in the diet was analyzed according to the methods described by the Association of Official Analytical Chemists (AOAC). SID Lys, Standard ileal digestible Lysine; SID Met, Standard ileal digestible Methionine; SID Thr, Standard ileal digestible Threonine; SID Trp, Standard ileal digestible Tryptophan.

**Supplementary Table S2** Distribution of samples

| Item | Batch 1 (NO. 1-5) | Batch 2 (NO. 6-11) | Total |
| --- | --- | --- | --- |
| Stomach | 5 | 6 | 11 |
| Duodenum | 4 | 6 | 10 |
| Jejunum | 5 | 3 | 8 |
| Ileum | 3 | 5 | 8 |
| Cecum | 5 | 5 | 10 |
| Colon | 5 | 6 | 11 |
| Total | 27 | 31 | 58 |

**Supplementary Table 3**  The number of assembled reads and other parameters for quality control of each sample

| Item | Raw reads | Filtered | Denoised | Non-chimeric | Percentage of input non-chimeric (%) |
| --- | --- | --- | --- | --- | --- |
| Sto1 | 80096 | 36251 | 36056 | 35974 | 44.91 |
| Sto2 | 80104 | 36300 | 36205 | 36105 | 45.07 |
| Sto3 | 80044 | 40320 | 40038 | 39866 | 49.81 |
| Sto4 | 46060 | 18708 | 18543 | 18543 | 40.26 |
| Sto5 | 80126 | 37779 | 37564 | 37181 | 46.4 |
| Sto6 | 82739 | 42414 | 42081 | 38446 | 46.47 |
| Sto7 | 81050 | 28580 | 28408 | 27304 | 33.69 |
| Sto8 | 82353 | 26625 | 26470 | 25120 | 30.5 |
| Sto9 | 81081 | 57539 | 57319 | 55798 | 68.82 |
| Sto10 | 83010 | 50835 | 50736 | 46132 | 55.57 |
| Sto11 | 66744 | 27378 | 27259 | 26981 | 40.42 |
| Duo2 | 80122 | 34274 | 34163 | 33719 | 42.08 |
| Duo3 | 80036 | 47126 | 46639 | 44653 | 55.79 |
| Duo4 | 80105 | 45838 | 45510 | 45297 | 56.55 |
| Duo5 | 80112 | 41797 | 41538 | 41059 | 51.25 |
| Duo6 | 73604 | 34103 | 34032 | 33670 | 45.74 |
| Duo7 | 81543 | 47736 | 47592 | 41781 | 51.24 |
| Duo8 | 83297 | 58163 | 57737 | 55704 | 66.87 |
| Duo9 | 81583 | 49178 | 48894 | 47745 | 58.52 |
| Duo10 | 83396 | 46414 | 46317 | 41560 | 49.83 |
| Duo11 | 83056 | 56468 | 56154 | 55207 | 66.47 |
| Jej1 | 39347 | 17446 | 17317 | 17146 | 43.58 |
| Jej2 | 68814 | 32836 | 32607 | 30701 | 44.61 |
| Jej3 | 55045 | 30900 | 30684 | 30236 | 54.93 |
| Jej4 | 73094 | 36585 | 36510 | 35375 | 48.4 |
| Jej5 | 69048 | 30191 | 30086 | 30086 | 43.57 |
| Jej7 | 81944 | 38882 | 38750 | 38616 | 47.12 |
| Jej9 | 65993 | 23259 | 23031 | 23031 | 34.9 |
| Jej10 | 21941 | 10643 | 10475 | 10475 | 47.74 |
| Ile2 | 35656 | 18232 | 17909 | 17779 | 49.86 |
| Ile3 | 59070 | 44325 | 44154 | 40259 | 68.15 |
| Ile5 | 47572 | 20631 | 20427 | 20193 | 42.45 |
| Ile6 | 53212 | 20078 | 19966 | 19966 | 37.52 |
| Ile7 | 80089 | 57016 | 56663 | 53030 | 66.21 |
| Ile8 | 81906 | 28689 | 28602 | 26713 | 32.61 |
| Ile10 | 83017 | 61350 | 61009 | 59667 | 71.87 |
| Ile11 | 80767 | 42845 | 42738 | 42738 | 52.92 |
| Cec1 | 57056 | 39861 | 39474 | 38443 | 67.38 |
| Cec2 | 79497 | 34908 | 34682 | 34177 | 42.99 |
| Cec3 | 80062 | 42690 | 42594 | 40438 | 50.51 |
| Cec4 | 72976 | 28510 | 28385 | 28033 | 38.41 |
| Cec5 | 80107 | 43245 | 42981 | 40863 | 51.01 |
| Cec7 | 69846 | 30426 | 30250 | 30165 | 43.19 |
| Cec8 | 82149 | 60553 | 60232 | 59366 | 72.27 |
| Cec9 | 82144 | 56681 | 56479 | 55977 | 68.14 |
| Cec10 | 81917 | 29330 | 28967 | 28892 | 35.27 |
| Cec11 | 64177 | 30857 | 30691 | 30587 | 47.66 |
| Col1 | 80120 | 68132 | 67807 | 67125 | 83.78 |
| Col2 | 80113 | 55877 | 55738 | 55552 | 69.34 |
| Col3 | 80096 | 66184 | 66059 | 64683 | 80.76 |
| Col4 | 87790 | 48578 | 48348 | 44586 | 50.79 |
| Col5 | 80113 | 52793 | 52659 | 52342 | 65.34 |
| Col6 | 82733 | 61358 | 60874 | 59738 | 72.21 |
| Col7 | 81795 | 38626 | 38490 | 37947 | 46.39 |
| Col8 | 82106 | 54118 | 53783 | 53232 | 64.83 |
| Col9 | 84451 | 49673 | 49420 | 47645 | 56.42 |
| Col10 | 82832 | 65974 | 65642 | 57620 | 69.56 |
| Col11 | 57916 | 26781 | 26632 | 26632 | 45.98 |

Sto: Stomach, Duo: Duodenum, Jej: Jejunum, Ile: Ileum, Cec: Cecum, Col: Colon. Numbers 1-5 belong to the Batch 1, 6-11 belong to the Batch 2.

**Supplementary Table 4**  The α-diversity of fungal community in the different segments of gastrointestinal digesta samples of pigs

| Group | Item | GI Tract | | | | | | *P* value |
| --- | --- | --- | --- | --- | --- | --- | --- | --- |
|  |  | Stomach | Duodenum | Jejunum | Ileum | Cecum | Colon |  |
| Batch 1 | Richness | 58±8 | 176±55 | 94±27 | 111±31 | 104±28 | 64±9 | 0.097 |
|  | Shannon | 0.98±0.19^a^ | 2.86±0.54^c^ | 1.62±0.38^ab^ | 2.21±0.59^bc^ | 1.70±0.22^ab^ | 1.29±0.19^ab^ | 0.014 |
|  | Chao 1 | 58.59±8.59 | 178.23±55.60 | 96.34±28.41 | 111.53±31.35 | 106.73±28.42 | 70.81±10.08 | 0.116 |
|  | Good’s coverage | 0.989±0.002 | 0.987±0.004 | 0.987±0.002 | 0.987±0.000 | 0.989±0.001 | 0.992±0.002 | 0.554 |
| Batch 2 | Richness | 136±28 | 143±43 | 148±34 | 170±38 | 190±35 | 228±27 | 0.392 |
|  | Shannon | 2.04±0.51 | 1.74±0.60 | 2.01±0.60 | 1.70±0.30 | 1.65±0.25 | 3.12±0.43 | 0.219 |
|  | Chao 1 | 154.48±30.69 | 171.78±45.42 | 160.14±38.61 | 196.28±36.23 | 230.34±42.59 | 264.68±37.00 | 0.306 |
|  | Good’s coverage | 0.991±0.001 | 0.992±0.001 | 0.992±0.001 | 0.992±0.001 | 0.990±0.001 | 0.990±0.001 | 0.723 |

Data are presented as means ± standard error (SEM). Different alphabetical (a, b, c) superscripts mean significant difference (*p* < 0.05).

**Supplementary Table 5 The relative abundance (%) of the main fungal phyla and genus in the different segments of gastrointestinal digesta samples of pigs (Batch 1)**

| Phylum | Different segments of gastrointestinal tract in pigs | | | | | | |
| --- | --- | --- | --- | --- | --- | --- | --- |
|  | Stomach | Duodenum | Jejunum | Ileum | Cecum | Colon | *p* value |
| Basidiomycota | 2.934±0.662^a^ | 9.172±3.920^ab^ | 41.740±16.972^bc^ | 39.288±14.052^bc^ | 66.884±13.650^cd^ | 91.249±3.087^d^ | 0.001 |
| Ascomycota | 5.606±1.451^ab^ | 28.421±9.683^c^ | 7.286±2.778^ab^ | 19.245±8.239^b^c | 5.755±1.756^ab^ | 3.502±0.676^a^ | 0.047 |
| Mucoromycota | 0.004±0.004 | 0.035±0.035 | 0.233±0.132 | 1.351±0.695 | 0.319±0.129 | 0.433±0.183 | 0.059 |
| Mortierellomycota | 0.233±0.181^a^ | 9.231±5.056^b^ | 1.102±0.310^a^ | 1.388±0.457^a^ | 1.109±0.388^a^ | 0.109±0.089^a^ | 0.025 |
| Rozellomycota | 0.127±0.060 | 0.110±0.039 | 0.834±0.819 | 0.020±0.020 | 0.011±0.007 | 0.002±0.002 | 0.268 |
| Olpidiomycota | 0.022±0.021 | 0.147±0.075 | 0.433±0.388 | 0.033±0.033 | 0.033±0.014 | 0.009±0.008 | 0.406 |
| Chytridiomycota | 0.246±0.084 | 0.246±0.133 | 0.002±0.001 | 0.002±0.002 | 0.050±0.030 | 0.031±0.014 | 0.111 |
| Aphelidiomycota | 0.021±0.015 | 0.028±0.022 | 0.104±0.104 | 0.012±0.012 | 0.009±0.004 | 0 | 0.606 |
| Others | 90.807±2.190^c^ | 52.610±17.458^bc^ | 48.266±17.776^b^ | 38.661±21.483^ab^ | 25.831±314.076^ab^ | 4.664±2.631^a^ | 0.007 |
| Genus | Stomach | Duodenum | Jejunum | Ileum | Cecum | Colon | *p* value |
| *Naganishia* | 2.101±0.314^a^ | 2.844±0.926^a^ | 33.635±15.061^ab^ | 28.171±8.624^ab^ | 54.901±13.910^bc^ | 80.202±9.326^c^ | 0.001 |
| *Rhodotorula* | 0.321±0.063^a^ | 0.287±0.167^a^ | 5.102±3.812^ab^ | 5.202±4.511^ab^ | 9.449±8.217^b^ | 9.713±7.568^b^ | 0.014 |
| *Fusarium* | 2.571±0.645 | 6.006±1.817 | 2.194±0.725 | 8.424±5.559 | 1.818±0.800 | 2.207±0.398 | 0.184 |
| *Mortierella* | 0.233±0.181^a^ | 3.651±2.282^b^ | 0.900±0.333^ab^ | 1.300±0.461^ab^ | 1.059±0.384^ab^ | 0.098±0.078^a^ | 0.035 |
| *Candida* | 0.114±0.040^ab^ | 0.476±0.259^ab^ | 1.509±1.148^ab^ | 1.822±1.039^b^ | 0.147±0.078^ab^ | 0.011±0.009^a^ | 0.033 |
| *Pseudombrophila* | 0 | 3.062±3.062 | 0 | 0 | 0.033±0.033 | 0 | 0.508 |
| *Preussia* | 0.045±0.029 | 2.206±1.338 | 0.051±0.033 | 0.194±0.157 | 0.138±0.138 | 0 | 0.212 |
| *Trichosporon* | 0.045±0.045 | 0.044±0.017 | 0.558±0.456 | 1.841±1.683 | 0.181±0.098 | 0.120±0.041 | 0.173 |
| *Tausonia* | 0.016±0.011 | 2.210±2.171 | 0.094±0.039 | 0.102±0.067 | 0.047±0.033 | 0.012±0.012 | 0.269 |
| *Apiotrichum* | 0.038±0.012 | 0.032±0.015 | 0.579±0.544 | 1.122±1.089 | 0.207±0.124 | 0.439±0.301 | 0.744 |
| Others | 94.516±0.662^d^ | 82.243±5.598^cd^ | 55.379±17.784^bc^ | 51.824±16.898^bc^ | 32.052±16.898^ab^ | 7.199±3.169^a^ | 0.002 |

Notes: Data are presented as mean ± SEM, different alphabetical (a, b, c, ab) superscripts mean significant difference (*p* < 0.05). “Others” means those phylum/genera could not be identified into any known phylum/genera. We only list the main genera with an average abundance of more than 1.00%.

**Supplementary Table 6 The relative abundance (%) of the main fungal phyla and genus in the different segments of gastrointestinal digesta samples of pigs (Batch 2)**

| Phylum | Different segments of gastrointestinal tract in pigs | | | | | | |
| --- | --- | --- | --- | --- | --- | --- | --- |
|  | Stomach | Duodenum | Jejunum | Ileum | Cecum | Colon | *p* value |
| Ascomycota | 43.546±9.099 | 34.511±13.525 | 41.345±15.421 | 71.891±15.356 | 50.388±17.336 | 38.385±10.325 | 0.373 |
| Basidiomycota | 8.988±4.249 | 9.745±7.009 | 3.046±1.707 | 3.546±1.451 | 3.468±0.882 | 16.760±7.523 | 0.238 |
| Mucoromycota | 0.846±0.417^a^ | 0.732±0.397^a^ | 1.627±1.035^ab^ | 1.662±0.631^ab^ | 5.400±2.934^ab^ | 7.178±2.581^b^ | 0.013 |
| Mortierellomycota | 0.872±0.645 | 0.277±0.165 | 1.114±0.981 | 0.632±0.362 | 0.505±0.243 | 2.607±1.661 | 0.783 |
| Rozellomycota | 0.022±0.016 | 0.128±0.128 | 0 | 0.076±0.073 | 0.032±0.029 | 0.016±0.009 | 0.808 |
| Glomeromycota | 0.016±0.009 | 0.002±0.002 | 0.159±0.116 | 0.039±0.018 | 0 | 0.053±0.032 | 0.081 |
| Others | 45.709±8.900 | 54.606±16.476 | 52.709±18.609 | 22.155±15.479 | 40.207±18.808 | 35.001±15.124 | 0.149 |
| Genus | Stomach | Duodenum | Jejunum | Ileum | Cecum | Colon | *p* value |
| *Kazachstania* | 22.045±12.721 | 14.086±12.617 | 27.835±13.915 | 52.452±15.538 | 41.796±17.775 | 23.833±10.535 | 0.520 |
| *Mucor* | 0.809±0.419^a^ | 0.618±0.305^a^ | 1.611±1.026^ab^ | 1.645±0.615^ab^ | 5.273±2.922^ab^ | 6.936±2.574^b^ | 0.010 |
| *Trichosporon* | 0.900±0.573 | 4.218±3.536 | 0.425±0.282 | 0.286±0.105 | 0.932±0.527 | 8.508±3.350 | 0.071 |
| *Nothophoma* | 0.352±0.231 | 0.063±0.045 | 0.077±0.077 | 11.027±10.829 | 0.295±0.173 | 0.813±0.748 | 0.371 |
| *Fusarium* | 2.983±1.453 | 1.338±0.621 | 1.643±0.421 | 1.142±0.416 | 1.650±0.338 | 2.105±0.697 | 0.829 |
| *Cladosporium* | 3.315±2.835 | 2.280±2.024 | 1.171±1.031 | 0.466±0.220 | 0.315±0.182 | 0.270±0.042 | 0.953 |
| *Cutaneotrichosporon* | 0.395±0.114 | 0.630±0.479 | 0.671±0.634 | 0.530±0.389 | 0.273±0.117 | 3.742±3.378 | 0.802 |
| *Schizophyllum* | 4.915±4.686 | 0.126±0.037 | 0.138±0.115 | 0.139±0.037 | 0.085±0.036 | 0.130±0.015 | 0.609 |
| *Mortierella* | 0.828±0.601 | 0.277±0.165 | 0.988±0.867 | 0.632±0.362 | 0.488±0.245 | 2.564±1.659 | 0.844 |
| *Huntiella* | 4.496±4.406 | 0.061±0.013 | 0.041±0.029 | 0.037±0.016 | 0.022±0.005 | 0.055±0.009 | 0.258 |
| Others | 58.964±10.245 | 76.303±12.660 | 65.410±16.171 | 31.535±16.554 | 48.870±18.525 | 51.043±13.275 | 0.311 |

Notes: Data are presented as mean ± SEM, different alphabetical (a, b, c, ab) superscripts mean significant difference (*p* < 0.05). “Others” means those phylum/genera could not be identified into any known phylum/genera. We only list the main genera with an average abundance of more than 1.00%.

**Supplementary Table 7**  Function annotation of fungi based on trophic mode and guild

| Trophic mode (%) | Batch 1 | | | | | | |
| --- | --- | --- | --- | --- | --- | --- | --- |
|  | Stomach | Duodenum | Jejunum | Ileum | Cecum | Colon | *p* value |
| Saprotroph | 1.989±0.885 | 18.169±7.890 | 5.988±2.421 | 13.790±5.296 | 3.970±1.066 | 1.952±0.514 | 0.012 |
| Pathotroph | 0.149±0.087 | 2.022±1.123 | 1.025±0.849 | 2.082±1.614 | 0.355±0.094 | 0.135±0.047 | 0.228 |
| Pathotroph-Saprotroph-Symbiotroph | 0.196±0.053 | 1.744±0.703 | 0.958±0.485 | 1.541±0.527 | 0.654±0.205 | 0.347±0.090 | 0.055 |
| Pathotroph-Symbiotroph | 0.054±0.021 | 0.291±0.166 | 0.085±0.029 | 0.126±0.044 | 0.006±0.005 | 0.011±0.003 | 0.047 |
| Pathotroph-Saprotroph | 2.939±0.633 | 6.626±1.795 | 7.386±4.317 | 13.822±6.305 | 11.306±8.998 | 11.926±7.890 | 0.826 |
| Symbiotroph | 0.029±0.025 | 0.659±0.315 | 0.167±0.099 | 0.355±0.173 | 0.482±0.302 | 0.066±0.060 | 0.180 |
| Pathogen-Saprotroph-Symbiotroph | 0.005±0.005 | 0.529±0.315 | 0.008±0.008 | 0.018±0.018 | 0.04±0.025 | 0.009±0.007 | 0.029 |
| Saprotroph-Symbiotroph | 0 | 0.26±0.155 | 0.002±0.002 | 0.102±0.039 | 0.008±0.008 | 0 | 0.021 |
| Unassigned | 94.678±1.166 | 69.698±10.965 | 84.381±7.661 | 68.165±12.924 | 83.179±9.146 | 85.554±8.370 | 0.303 |
| Trophic guild (%) | Stomach | Duodenum | Jejunum | Ileum | Cecum | Colon | *p* value |
| Animal_Endosymbiont-Undefined  _Saprotroph | 0.321±0.063 | 0.287±0.167 | 5.102±3.812 | 5.296±4.464 | 9.458±8.215 | 9.713±7.568 | 0.708 |
| Undefined_Saprotroph | 1.934±0.885^a^ | 16.729±7.358^c^ | 5.182±1.816^ab^ | 12.214±5.258^bc^ | 3.267±0.995^ab^ | 1.411±0.433^a^ | 0.012 |
| Plant_Pathogen-Soil_Saprotroph-Wood  _Saprotroph | 2.571±0.645 | 6.006±1.817 | 2.194±0.725 | 8.424±5.559 | 1.818±0.799 | 2.207±0.398 | 0.184 |
| Animal_Pathogen | 0.048±0.044 | 0.222±0.126 | 0.560±0.459 | 1.841±1.682 | 0.216±0.087 | 0.120±0.041 | 0.236 |
| Animal_Pathogen-Endophyte  -Plant_Pathogen-Wood_Saprotroph | 0.140±0.049^a^ | 0.816±0.248^b^ | 0.124±0.061^a^ | 0.167±0.035^a^ | 0.087±0.036^a^ | 0.033±0.011^a^ | 0.038 |
| Plant_Pathogen | 0.056±0.051 | 1.546±0.991 | 0.032±0.013 | 0.125±0.079 | 0.106±0.058 | 0.006±0.003 | 0.085 |
| Soil_Saprotroph | 0.045±0.013 | 0.349±0.300 | 0.754±0.648 | 1.477±1.096 | 0.625±0.091 | 0.533±0.287 | 0.258 |
| Ectomycorrhizal | 0.020±0.016 | 0.344±0.204 | 0.146±0.102 | 0.255±0.123 | 0.445±0.306 | 0.059±0.053 | 0.375 |
| Plant_Pathogen_Wood_Saprotroph | 0.076±0.032^a^ | 0.287±0.168^b^ | 0.084±0.030^a^ | 0.077±0.029a | 0.005±0.005^a^ | 0.007±0.003^a^ | 0.008 |
| Animal_Parasite-Fungal_Parasite | 0.022±0.021 | 0.147±0.075 | 0.433±0.388 | 0.033±0.033 | 0.033±0.014 | 0.009±0.008 | 0.502 |
| Unassigned | 94.638±1.166 | 69.698±10.965 | 84.381±7.661 | 68.165±12.924 | 83.179±9.146 | 85.554±8.370 | 0.303 |

Notes: Only the confidence values considered high probability and probable were counted as known trophic modes or guilds. Cases in which confidence was possible and undefined were defined as unknown trophic modes. Guilds were filtered by the criteria of mean relative abundance greater than 0.1% in at least one of all samples.

**Supplementary Table 8**  Function annotation of fungi based on trophic mode and guild

| Trophic guild (%) | Batch 2 | | | | | | |
| --- | --- | --- | --- | --- | --- | --- | --- |
|  | Stomach | Duodenum | Jejunum | Ileum | Cecum | Colon | *p* value |
| Saprotroph | 34.801±10.112 | 26.769±13.403 | 35.928±14.762 | 58.816±15.176 | 52.264±18.674 | 41.148±11.561 | 0.602 |
| Pathotroph | 6.454±4.227 | 6.001±4.657 | 3.656±3.057 | 0.881±0.402 | 1.376±0.446 | 9.226±3.473 | 0.513 |
| Pathotroph-Saprotroph-Symbiotroph | 1.053±0.541 | 3.473±2.991 | 1.029±0.653 | 1.057±0.567 | 0.813±0.434 | 1.478±0.900 | 0.807 |
| Pathotroph-Symbiotroph | 3.439±2.814 | 2.308±2.020 | 1.171±1.031 | 0.471±0.223 | 0.359±0.202 | 0.279±0.048 | 0.669 |
| Pathotroph-Saprotroph | 3.132±1.572 | 2.316±0.960 | 1.818±0.488 | 1.469±0.558 | 1.823±0.360 | 2.312±0.708 | 0.870 |
| Symbiotroph | 0.136±0.089 | 0.134±0.068 | 0.260±0.124 | 0.146±0.055 | 0.105±0.054 | 0.451±0.202 | 0.265 |
| Pathogen-Saprotroph-Symbiotroph | 0.144±0.137 | 0.106±0.058 | 0.098±0.060 | 0.078±0.026 | 0.024±0.013 | 0.356±0.163 | 0.311 |
| Saprotroph-Symbiotroph | 0.132±0.079 | 0.083±0.031 | 0.199±0.169 | 0.093±0.056 | 0.159±0.123 | 0.020±0.013 | 0.694 |
| Unassigned | 50.707±8.681 | 58.809±15.344 | 55.840±17.688 | 36.988±15.136 | 43.070±18.501 | 44.728±12.866 | 0.901 |
| Trophic guild (%) | Stomach | Duodenum | Jejunum | Ileum | Cecum | Colon | *p* value |
| Undefined_Saprotroph | 28.977±11.643 | 26.135±13.317 | 34.883±14.278 | 58.411±15.144 | 51.967±18.635 | 40.371±11.550 | 0.537 |
| Animal_Pathogen | 0.903±0.572 | 4.260±3.536 | 0.435±0.271 | 0.298±0.111 | 0.991±0.512 | 8.859±3.352 | 0.109 |
| Plant_Pathogen | 5.409±4.357 | 1.623±1.059 | 3.217±2.789 | 0.556±0.280 | 0.322±0.071 | 0.665±0.151 | 0.841 |
| Wood_Saprotroph | 5.004±4.668 | 0.350±0.209 | 0.142±0.112 | 0.320±0.144 | 0.102±0.041 | 0.148±0.028 | 0.149 |
| Endophyte-Plant_Pathogen | 3.420±2.816 | 2.302±2.021 | 1.171±1.031 | 0.471±0.223 | 0.349±0.202 | 0.272±0.044 | 0.673 |
| Animal_Pathogen-Endophyte-Plant  _Pathogen-Wood_Saprotroph | 0.510±0.287 | 2.113±1.793 | 0.732±0.570 | 0.544±0.310 | 0.347±0.226 | 0.647±0.495 | 0.886 |
| Plant_Pathogen-Soil  _Saprotroph-Wood_Saprotroph | 2.983±1.453 | 1.338±0.621 | 1.643±0.421 | 1.142±0.416 | 1.650±0.338 | 2.105±0.697 | 0.829 |
| Fungal_Parasite-Undefined_Saprotroph | 0.516±0.269 | 1.265±1.135 | 0.199±0.103 | 0.378±0.242 | 0.386±0.209 | 0.781±0.437 | 0.917 |
| Animal_Endosymbiont  -Undefined_Saprotroph | 0.102±0.042 | 0.476±0.420 | 0.195±0.171 | 0.205±0.110 | 0.112±0.095 | 0.026±0.016 | 0.407 |
| Soil_Saprotroph | 0.384±0.222 | 0.057±0.055 | 0.525±0.445 | 0.044±0.033 | 0.129±0.114 | 0.464±0.294 | 0.195 |
| Unassigned | 50.707±8.681 | 58.809±15.344 | 55.840±17.688 | 36.988±15.136 | 43.069±18.501 | 44.728±12.866 | 0.901 |

Notes: Only the confidence values considered high probability and probable were counted as known trophic modes or guilds. Cases in which confidence was possible and undefined were defined as unknown trophic modes. Guilds were filtered by the criteria of mean relative abundance greater than 0.1% in at least one of all samples.

**Supplementary Figure 1** Rarefaction curves based on alpha diversity index were used to assess the depth of coverage for each sample. Each sample was distinguished by different colors of lines. Sto: Stomach, Duo: Duodenum, Jej: Jejunum, Ile: Ileum, Cec: Cecum, Col: Colon.

**Supplementary Figure 2** Comparison of fungal diversity in the GIT of growing-finishing pigs with principal coordinate analysis (PCoA). The PCoA plot was generated using unweighted UniFrac. Sto: Stomach, Duo: Duodenum, Jej: Jejunum, Ile: Ileum, Cec: Cecum, Col: Colon.

**Supplementary Figure 3** Comparison of fungal diversity in the GIT of growing-finishing pigs with principal coordinate analysis (PCoA). The PCoA plot was generated using Bray-Curtis. (**A**) Batch 1, (**B**) Batch 2. Sto: Stomach, Duo: Duodenum, Jej: Jejunum, Ile: Ileum, Cec: Cecum, Col: Colon.

**Supplementary Figure 4** Fungal composition in the GI tract of growing-finishing pigs from two batches. (**A-B**) Phylum level, (**C-D**) Genus level. Only the known phyla and top 10 genera (the average abundance of more than 1.00%) are shown, with unidentified fungal taxa and those low-abundance taxa being collectively denoted as “Others.” Each bar represents the relative abundance of each fungal taxon in the corresponding sample. Sto: Stomach; Duo: Duodenum; Jej: Jejunum; Ile: Ileum; Cec: Cecum; Col: Colon.
